# Supplementary material for: Hypermethylation Loci of ZNF671, IRF8, and OTX1 as Potential Urine-Based Predictive Biomarkers for Bladder Cancer
Source: Diagnostics (Basel). 2024 Feb 21;14(5):468. doi: 10.3390/diagnostics14050468 (PMC10931331; doi:10.3390/diagnostics14050468)
Supplement: Supplementary file 1 [file diagnostics-14-00468-s001.zip › Supplementary File_Figure S1_hjw_rev.pdf]

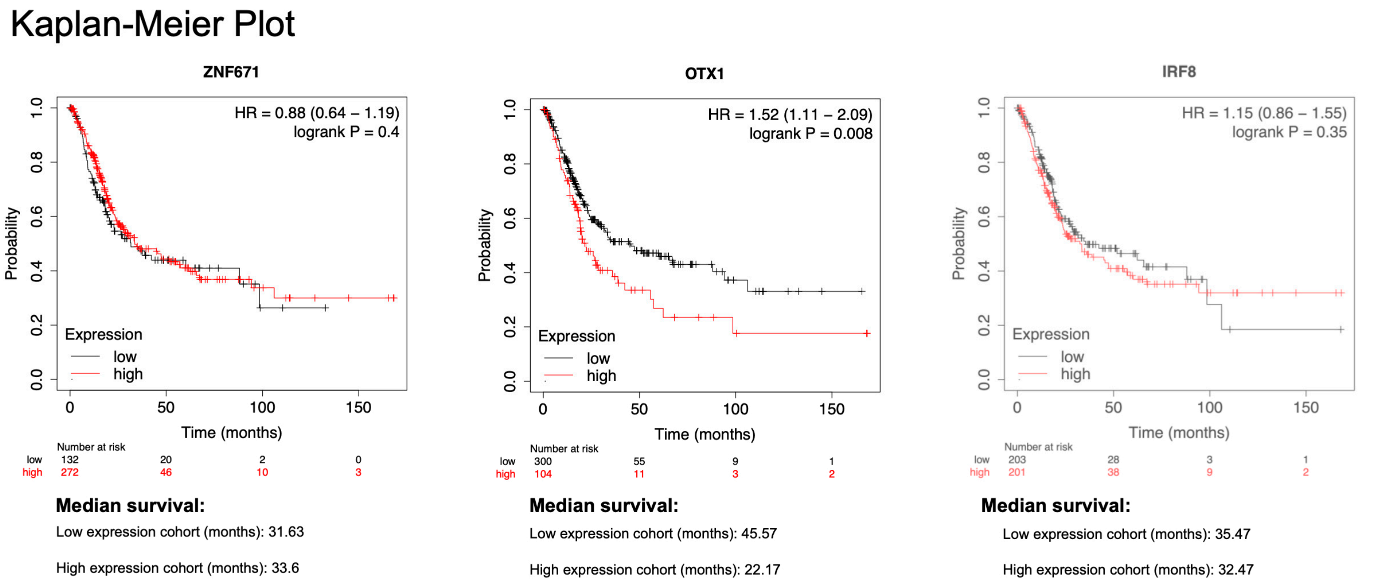

Figure S1. Survival curves of ZNF671, OTX1, and IRF8. The survival analysis of the three genes is based on their expression levels and utilizes the Kaplan-Meier method to generate survival curves. The graphics are generated by using the web server https://kmplot.com/analysis/ (accessed on 7 February 2024).
