# Supplementary material for: Hypermethylation Loci of ZNF671, IRF8, and OTX1 as Potential Urine-Based Predictive Biomarkers for Bladder Cancer
Source: Diagnostics (Basel). 2024 Feb 21;14(5):468. doi: 10.3390/diagnostics14050468 (PMC10931331; doi:10.3390/diagnostics14050468)
Supplement: Supplementary file 1 [file diagnostics-14-00468-s001.zip › Supplementary File_Table S1_hjw_rev.pdf]

Supplementary Table S1. Primer sequences, annealing temperatures, and amplicon information for the qMSP assay used in the study

Primer name	Sequence	Tm (°C)	Amplicon (bp)
ZNF671_F	5'-TTTGGTCGGGGTTTTGTAAC-3'	62.4	100
ZNF671_R	5'-AACATCAAACGCGTCTCG -3'	61.4	
OTX1_F	5'-TCGATTTTGTTTTACGTTTGTC -3'	60.9	109
OTX1_R	5'-CTAAAAACGCGCTCCTCC -3'	62.9	
IRF8_F	5'-ATTTTCGGGGTTGTTCGTTC 3'	63.7	120
IRF8_R	5'-CACCTAAAATCCAAAAACGACG -3'	63.1	
COL2A1_F	5'-GGGAAGATGGGATAGAAGGGAATAT -3'	65.8	89
COL2A1_F	5'-AACAATTATAAACTCCAACCAC -3'	56.2	
